# Successful Percutaneous Coronary Intervention through a Severely Bent Artificial Ascending Aorta Using the DIO Thrombus Aspiration Catheter

**DOI:** 10.1155/2016/8502356

**Published:** 2016-07-19

**Authors:** Akinori Fujikake, Takaaki Komatsu, Isao Taguchi

**Affiliations:** Department of Cardiology, Dokkyo Medical University Koshigaya Hospital, 2-1-50 Minami Koshigaya, Koshigaya City, Saitama 3438555, Japan

## Abstract

A 66-year-old man was admitted to our institute because of chest pain. He had undergone replacement of the ascending aorta due to aortic dissection 9 years previously. We made a diagnosis of acute coronary syndrome, and coronary artery angiography was performed. Although the right coronary artery was successfully cannulated, a severe bend of the artificial aorta made it very difficult to advance the catheter into the left coronary artery. Ultimately, a DIO thrombus aspiration catheter was used to enter the left coronary artery, and a stent was implanted successfully. The DIO catheter is very useful when the selection of a guiding catheter is complicated, such as in the case of severe vessel tortuosity or a bend of the ascending aorta.

## 1. Introduction

Coronary artery disease is often accompanied by other vascular diseases. Selection of an appropriate guiding catheter is the most important determinant of procedural success in percutaneous coronary intervention (PCI). However, in cases of patients with diseases of the ascending aorta, it may be difficult to advance the guiding catheter into a coronary artery. We report a successful case of PCI performed through a severe bend of an artificial ascending aorta using the DIO thrombus aspiration catheter.

## 2. Case Series

**Figure 1:**
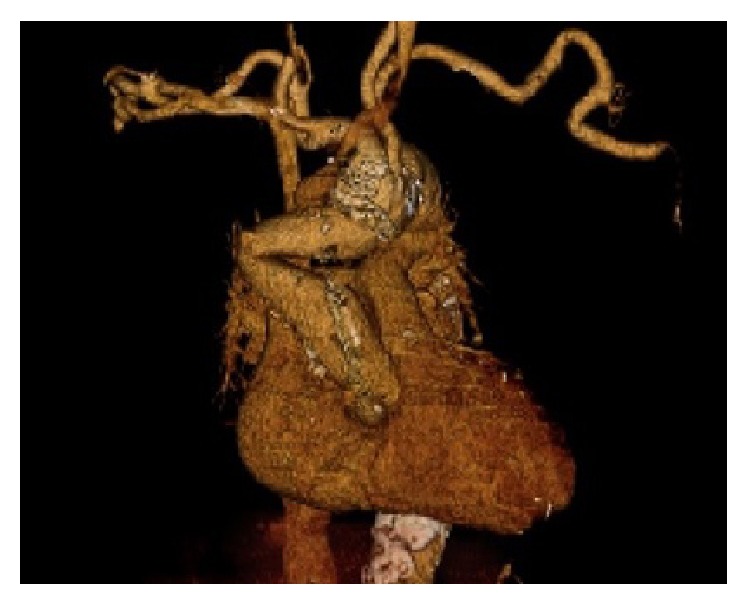
Chest 3-dimensional computed tomography. Computed tomography revealed that the replaced duct was severely bent in the ascending aorta.

A 66-year-old man was admitted to our institute because of chest pain. He had undergone replacement of the ascending aorta due to aortic dissection 9 years previously. His risk factors for coronary artery disease included hypertension and hemodialysis due to chronic nephrotic syndrome. An electrocardiogram taken on admission showed regular sinus rhythm with ST depression in leads V1–V5. Enhanced computed tomography of the chest revealed that the replaced artificial duct in the ascending aorta was severely bent (Figure 1). He was diagnosed with acute coronary syndrome and underwent coronary artery angiography (CAG) via a right femoral arterial approach. Selective cannulation of the right coronary artery was achieved with 4.2 Fr Judkins right 4.0 catheter. However, when selective cannulation of the left coronary artery was attempted, the catheter could not be controlled because of a severely bent duct in the ascending aorta. The left coronary artery could not be reached using a 4.2 Fr Judkins left 4.0 catheter, Judkins left 5.0 catheter, Amplatz 1.0 catheter, dual use catheter, or 6 Fr guiding catheter. As the next strategy, we first considered using a 4.2 Fr diagnostic catheter in a 6 Fr guiding catheter (mother-child technique). However, a 6 Fr guiding catheter could not advance beyond the severely bent part of the artificial ascending aorta due to its low profile. Next, we considered using a 4.2 Fr diagnostic catheter in a 5 Fr guiding catheter. However, we thought that a 4.2 Fr diagnostic catheter could not be controlled enough to engage the left coronary artery due to the unfavorable shape of the 5 Fr guiding catheter tip. In addition, the softer tip of DIO compared to 5 Fr guiding catheter seemed to be more suitable for this complex lesion which should need the deep engagement of guiding catheter.

**Figure 2:**
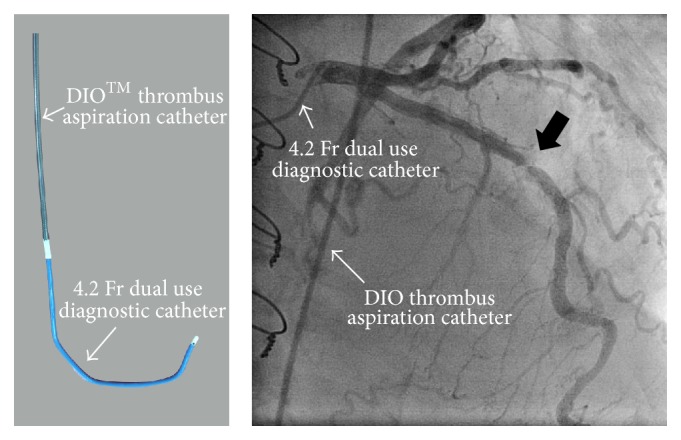
Left coronary artery angiography supported DIO thrombus aspiration catheter. A 4.2 Fr dual use catheter was inserted into the DIO thrombus aspiration catheter. Selective injection revealed 99% stenosis at the midportion of the left anterior descending artery.

**Figure 3:**
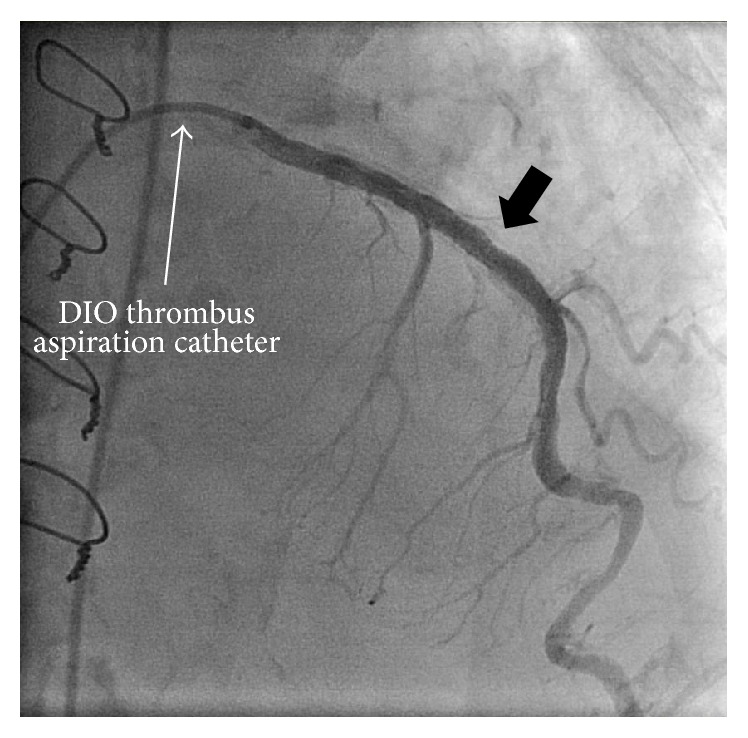
Coronary angiography after percutaneous coronary intervention. The residual stenosis was <5%.

Finally, we decided to insert a 4.2 Fr dual use catheter into a DIO thrombus aspiration catheter (a straight catheter) to pass the artificial duct in the ascending aorta. Then, alternate manipulation of the DIO thrombus aspiration catheter and the angiographic catheter was very effective in advancing the two catheters through the artificial aorta, allowing these two catheters to be inserted into the left coronary artery. Selective injection of contrast revealed 99% stenosis at the midportion of the left anterior descending (LAD) artery (Figure 2). After that, we advanced the DIO thrombus aspiration catheter forward deeply into the LAD artery, pulled out the angiographic catheter, and performed PCI using the DIO thrombus aspiration catheter as the guiding catheter. At this time, we inserted a 0.014 SION Blue guidewire (Asahi Intech Corp., Japan) through the 4.2 Fr diagnostic catheter into the coronary artery before pulling out the 4.2 Fr diagnostic catheter to prevent removal of the DIO from the coronary artery. IVUS evaluation was important for achieving optimal PCI to avoid the need for repeat PCI in this difficult case. We predilated the lesion with a 2.5 × 15 mm TREK Balloon Catheter (Abbott Vascular Co., Ltd., CA, USA) and engaged the DIO thrombus aspiration catheter into the LAD artery more deeply to gain more back-up support. Subsequently, an Eagle Eye IVUS Catheter (Eagle Eye: Volcano Corp., USA) was inserted to the midportion of the LAD artery. After that, stenting was accomplished with a 3.5 × 15 mm Xience Xpedition Stent (Abbott Vascular Co., Ltd., CA, USA) delivered at 14 atm. The residual stenosis was <5% (Figure 3). This excellent result was accomplished using the DIO thrombus aspiration catheter.

## 3. Discussion

After replacement of the ascending aorta, a severe bend in the aorta is a rare complication [1]. However, a severe bend in the ascending aorta with or without replacement can make it very difficult to perform PCI.

There are three possible benefits of using the DIO catheter to perform PCI. First, substituting a DIO catheter for a long sheath can make it easy to control a diagnostic catheter for cannulation. Next, substituting a DIO catheter for a guiding catheter can shorten the PCI procedure, because it is not necessary to exchange the diagnostic catheter for a guiding catheter. Finally, strong back-up support can be achieved by deep engagement of the DIO catheter.

Selecting an appropriate guiding catheter is the most important determinant of procedural success in PCI.However, in this case, it was not possible to reach the left coronary ostium, even with the diagnostic catheter. We previously reported successful PCI of an anomalous right coronary artery using the DIO thrombus aspiration catheter [2]. The DIO thrombus aspiration catheter that includes a 4.2 Fr angiographic catheter has become available. Therefore, it is possible to engage the coronary artery by sliding the angiographic catheter through the DIO catheter. A tortuosity or severe bend of the ascending aorta is a serious problem when PCI is performed. In the case of a severe bend of the ascending aorta, it is not uncommon to kink the angiographic catheter, and the DIO thrombus aspiration catheter can provide strong support of the catheter without kinking. By inserting the DIO thrombus aspiration catheter into the ascending aorta, it is possible to operate the angiographic catheter more delicately.

Sometimes, CAG followed by PCI becomes complex, and the duration of the procedure is prolonged because of the time required to select an adequate guiding catheter. By inserting a 4.2 Fr diagnostic catheter into the DIO thrombus aspiration catheter, it is possible to perform CAG and PCI consecutively and safely. Previously, the mother-child technique was useful in cases in which it was difficult to engage the guiding catheter or strong back-up was needed [3]. Recently, a GuideLiner® catheter (Vascular Solutions Inc., MN, USA) was used for the mother-child technique [4]. However, in the present case, we thought that this method would not be useful, because a guiding catheter could not be inserted through the severe bend in the ascending aorta. Therefore, the DIO thrombus aspiration catheter can be useful when selection of the guiding catheter is rather complicated, such as in the case of a severely bent aorta.

Strong back-up support is one of the important determinants of PCI success. Generally, interventionists obtain back-up support by selecting the most appropriate French size or shape of the guiding catheter. A 7 or 8 Fr guiding catheter is rigid and could not be manipulated well in the present case. The DIO thrombus aspiration catheter allows coronary angiography and engages the coronary artery coaxially because of its softness. Moreover, strong back-up support can be obtained by deeply engaging the coronary artery. The DIO thrombus aspiration catheter allows balloon delivery, IVUS, and stenting of the distal portion of a vessel.

In the case of a severe bend of the ascending aorta, it is difficult to pass a diagnostic catheter and perform PCI. The DIO thrombus aspiration catheter is useful for PCI as well as diagnosis.

## 4. Conclusion

We report a successful case of PCI using a DIO thrombus aspiration catheter in a patient with a severe bend in the ascending aorta after aortic replacement. The DIO thrombus aspiration catheter is useful when selection of the guiding catheter is rather complicated, such as in the case of a severe bend of the ascending aorta.
